# Renal function in patients with significant tricuspid regurgitation: pathophysiological mechanisms and prognostic implications

**DOI:** 10.1111/joim.13312

**Published:** 2021-06-10

**Authors:** S. C. Butcher, F. Fortuni, M. F. Dietz, E. A. Prihadi, P. van der Bijl, N. Ajmone Marsan, J. J. Bax, V. Delgado

**Affiliations:** ^1^ From the Department of Cardiology Leiden University Medical Center Leiden The Netherlands; ^2^ Department of Cardiology Royal Perth Hospital Perth WA Australia; ^3^ Department of Molecular Medicine University of Pavia Pavia Italy; ^4^ Antwerp Cardiovascular Center ZNA Middelheim Antwerp Belgium

**Keywords:** chronic kidney disease, renal dysfunction, right ventricular dysfunction, tricuspid regurgitation

## Abstract

**Background:**

The pathophysiological mechanisms linking tricuspid regurgitation (TR) and chronic kidney disease (CKD) remain unknown. This study aimed to determine which pathophysiological mechanisms related to TR are independently associated with renal dysfunction and to evaluate the impact of renal impairment on long‐term prognosis in patients with significant (≥ moderate) secondary TR.

**Methods:**

A total of 1234 individuals (72 [IQR 63–78] years, 50% male) with significant secondary TR were followed up for the occurrence of all‐cause mortality and the presence of significant renal impairment (eGFR of <60 mL min^−1^ 1.73 m^−2^) at the time of baseline echocardiography.

**Results:**

Multivariable analysis demonstrated that severe right ventricular (RV) dysfunction (TAPSE < 14 mm) was independently associated with the presence of significant renal impairment (OR 1.49, 95% CI 1.11 to 1.99, *P* = 0.008). Worse renal function was associated with a significant reduction in survival at 1 and 5 years (85% vs. 87% vs. 68% vs. 58% at 1 year, and 72% vs. 64% vs. 39% vs. 19% at 5 years, for stage 1, 2, 3 and 4–5 CKD groups, respectively, *P* < 0.001). The presence of severe RV dysfunction was associated with reduced overall survival in stage 1–3 CKD groups, but not in stage 4–5 CKD groups.

**Conclusions:**

Of the pathophysiological mechanisms identified by echocardiography that are associated with significant secondary TR, only severe RV dysfunction was independently associated with the presence of significant renal impairment. In addition, worse renal function according to CKD group was associated with a significant reduction in survival.

## Graphical abstract

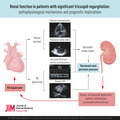

## Introduction

Secondary tricuspid regurgitation (TR), the principal mechanism of TR, is common, with a complex and often multifactorial aetiology including left‐sided valvular heart disease, pulmonary hypertension and left ventricular (LV) dysfunction [1]. Contemporary epidemiological studies have demonstrated that significant (≥ moderate) secondary TR is independently associated with poor long‐term prognosis [2, 3], which has led to significant interest in the development of tricuspid valve interventions that may modify this unfavourable natural history [4].

However, how TR contributes to increased mortality remains ill‐defined. Possibilities include acute or chronic right ventricular (RV) failure, acceleration of LV failure or reduced physiological reserve secondary to renal or hepatic impairment from chronically elevated central venous pressure. Indeed, several studies have demonstrated that worsening TR grade (ranging from none to severe) in individuals with heart failure is independently associated with renal dysfunction [2, 5], which could theoretically lead to increased rates of cardiovascular and non‐cardiovascular mortality as a consequence of chronic kidney disease (CKD) [6]. However, the pathophysiological mechanisms underlying the association between significant renal impairment and secondary TR remain unknown. In addition, the prognostic implications of renal impairment in a patient cohort with significant secondary TR have not yet been elucidated.

Therefore, the aim of this study was to (i) investigate the prevalence of renal impairment in individuals with significant secondary TR, (ii) determine the pathophysiological mechanisms identified by echocardiography that are associated with significant renal impairment in secondary TR and (iii) to investigate the prognostic implications of renal impairment in significant secondary TR.

## Materials and methods

### Study population

Patients diagnosed with moderate or severe secondary TR between June 1995 and September 2016 were selected from the departmental echocardiographic database at Leiden University Medical Center (Leiden, the Netherlands). Patients with congenital heart disease and those who underwent tricuspid valve repair were excluded. Additionally, patients with incomplete data to assess TR severity or without renal function recorded were excluded. Patient demographic and clinical data were obtained from the departmental electronic medical record (EPD‐vision; Leiden University Medical Center, Leiden, the Netherlands). As this study involved the retrospective analysis of clinically acquired data, the institutional review board of the Leiden University Medical Center waived the need for written patient informed consent. This investigation conforms to the principles outlined in the *Declaration of Helsinki*.

### Clinical and echocardiographic parameters

Clinical, demographic and laboratory variables were recorded from the time of first diagnosis of moderate or severe secondary TR by transthoracic echocardiography. Estimated glomerular filtration rate (eGFR) was calculated according to the Modification of Diet in Renal Disease formula [7]. Patients were subsequently divided into four categories according to eGFR, as per the recommendations of contemporary guidelines [7]. These groups were defined as normal renal function (stage 1 CKD, eGFR ≥90 mL min^−1^ 1.73 m^−2^), mildly impaired renal function (stage 2 CKD, eGFR 60 to 89 mL min^−1^ 1.73 m^−2^), moderately impaired renal function (stage 3 CKD, eGFR 30 to 59 mL min^−1^ 1.73 m^−2^) and severely impaired renal function (stage 4 and 5 CKD, eGFR <30 mL min^−1^ 1.73 m^−2^). Patients with an eGFR of <60 mL min^−1^ per 1.73 m^2^ were defined as having significant renal impairment [7].

Comprehensive transthoracic echocardiography was performed with patients at rest in the left lateral decubitus position, using Vivid 7, E9 and E95 ultrasound systems (General Electric Vingmed Ultrasound) equipped with 3.5 MHz or M5S transducers. All echocardiographic data were stored digitally in a cine‐loop format for offline analysis with EchoPac software (EchoPAC version 113.0.3, 202 and 203; GE‐Vingmed). Apical, parasternal and subcostal views were used to acquire M‐mode, 2‐dimensional and colour‐, continuous‐ and pulsed‐wave Doppler data according to contemporary guideline recommendations [8]. LV end‐diastolic and end‐systolic volumes were calculated using the biplane Simpson method and used to derive the LV ejection fraction. LV mass was calculated using the 2‐dimensional linear approach [9]. Significant (moderate or severe) mitral regurgitation and aortic stenosis were defined according to contemporary guidelines [8]. TR grade was evaluated using a multiparametric approach according to guideline recommendations, integrating qualitative, semiquantitative and quantitative parameters [10]. Pacemaker or implantable cardioverter–defibrillator lead‐related TR was only classified as primary TR in the absence of significant left‐sided valvular heart disease (defined as ≥ moderate mitral regurgitation/stenosis or aortic stenosis/regurgitation) or LV myocardial disease (defined as LV ejection fraction < 50%). RV dimensions, RV end‐systolic and RV end‐diastolic areas were acquired using an RV‐focused apical view. Tricuspid annular plane systolic excursion (TAPSE) was used to quantify RV systolic function, derived from M‐mode recordings of the lateral tricuspid annulus in an RV‐focused apical view. Severe RV dysfunction was defined by a TAPSE <14 mm [11]. Pulmonary artery systolic pressure was estimated by applying the modified Bernoulli equation to the TR jet peak velocity and adding mean right atrial (RA) pressure. Estimated RA pressure was calculated from the inferior vena cava diameter and its collapsibility. All other standard echocardiographic measurements were performed according to the American Society of Echocardiography and European Association of Cardiovascular Imaging guidelines [9].

### Follow‐up

All patients were followed up for the endpoint of all‐cause mortality. Survival data were collected through the Social Security Death Index or by medical record review and were complete for all patients. Follow‐up began from the date of first diagnosis of moderate or severe TR by transthoracic echocardiography.

### Statistical analysis

Categorical variables are expressed as numbers and percentages and were compared using the Pearson chi‐square test. Assessment of the distribution of continuous variables was performed by comparing a histogram of the sample data with a superimposed normal probability curve. Normally distributed continuous variables are presented as mean ± standard deviation, while variables that are non‐normally distributed are displayed as median and interquartile range. Differences between the four renal function groups were analysed using one‐way ANOVA for continuous variables that were normally distributed, while the Kruskal–Wallis test was used to compare continuous variables that did not adhere to a normal distribution. Multiple comparisons for continuous and categorical variables were tested using Bonferroni’s correction.

To investigate the association between clinical and echocardiographic parameters with the presence of significant renal impairment, univariable and multivariable logistic regression analyses were performed. Clinically important variables known or postulated to be associated with significant renal impairment [12, 13, 14] and with a *P*‐value <0.05 on univariable analysis were included in the multivariable model. A minimum tolerance level of 0.5 was established to avoid multicollinearity between covariates. To further characterize the relationship of RV systolic function (i.e. TAPSE) and the probability of significant renal impairment, a spline curve was fitted in unadjusted and adjusted models. A sensitivity analysis using multivariable logistic regression was performed to investigate the relationship between clinical and echocardiographic parameters and severely impaired renal function. An additional sensitivity analysis using univariable and multivariable linear regression was performed to examine the association between clinical and echocardiographic parameters with eGFR as a continuous variable. Cumulative 1‐ and 5‐year survival rates were calculated using the Kaplan–Meier method, and differences between groups were analysed using the log‐rank test.

All tests were two‐sided, and *P*‐values <0.05 were considered statistically significant. Statistical analysis was performed using the SPSS version 25.0 (IBM Corporation, Armonk, NY, USA) and R version 4.0.1 (R Foundation for Statistical Computing, Vienna, Austria).

## Results

### Clinical and echocardiographic characteristics

A total of 1234 patients with significant secondary TR were included. The median age of the population was 72 (interquartile range 63–78) years, 50% were male, and 23% had severe TR. The potential contributing aetiologies of secondary TR in the overall population included the following: LV ejection fraction <40% (41.1%), atrial fibrillation (49.5%), significant mitral regurgitation (27.4%), significant aortic stenosis (21.2%), chronic obstructive pulmonary disease (14.4%) and pulmonary hypertension (defined as a pulmonary artery systolic pressure >40 mmHg) (53.9%). The population was divided into four groups based on renal function: 230 (18.6%) had normal renal function, 451 (36.6%) had mildly impaired renal function, 439 (35.6%) had moderately impaired renal function, while 114 (9.2%) had severely impaired renal function. Those with renal impairment were older and more frequently hypertensive when compared to those with normal renal function. When compared to those with normal or mildly impaired renal function, patients with moderately or severely impaired renal function had more diabetes mellitus, coronary artery disease and peripheral oedema, were more often prescribed diuretics and presented more frequently with New York Heart Association III or IV heart failure symptoms. The baseline clinical characteristics of the population are summarized in Table 1.

**Table 1:** Clinical and demographic characteristics

Variable	Total population (*n* = 1234)	Group 1: GFR ≥ 90 mL min^−1^ 1.73 m^−2^ (*n* = 230)	Group 2: GFR 60–89 mL min^−1^ 1.73 m^−2^ (*n* = 451)	Group 3: GFR 30–59 mL min^−1^ 1.73 m^−2^ (*n* = 439)	Group 4: GFR < 30 mL min^−1^ 1.73 m^−2^ (*n* = 114)	*P* value
Age (years)	72 (63–78)	65 (55–75)	72 (63–79)*	74 (67–80)*	71 (64–78)*	<0.001
Male sex (%)	612 (49.6%)	124 (53.9%)	217 (48.1%)	218 (49.7%)	53 (46.5%)	0.466
Body mass index (kg/m^2^)	25.6 (±4.3)	25.1 (±4.3)	25.7 (±4.2)	25.6 (±4.2)	26.4 (±4.6)	0.129
Hypertension (%)	929 (80.2%)	147 (66.8%)	336 (80.6%)*	353 (84.7%)*	93 (88.6%)*	<0.001
Dyslipidaemia (%)	550 (47.6%)	88 (40.4%)^a^	180 (43.3%)	225 (54.0%)* ^,^ ^†^	57 (54.3%)	0.001
Diabetes mellitus (%)	238 (20.5%)	32 (14.6%)	55 (13.2%)	100 (23.9%)*,^†^	51 (48.1%)*,^†^,^§^	<0.001
Coronary artery disease (%)	498 (40.4%)	62 (27.0%)	154 (34.1%)	215 (49.1%)* ^,^ ^†^	67 (58.8%)*,^†^	<0.001
Chronic obstructive pulmonary disease (%)	168 (14.4%)	24 (10.9%)	54 (12.8%)	70 (16.7%)	20 (18.7%)	0.086
Current or former smoker (%)	365 (31.6%)	73 (33.5%)	138 (33.3%)	127 (30.6%	27 (25.5%)	0.405
Atrial fibrillation (%)	586 (49.5%)	94 (42.0%)	221 (51.5%)	222 (52.4%)	49 (45.4%)	0.048
NYHA class III‐IV (%)	534 (47.3%)	82 (40.6%)	168 (40.7%)	211 (51.8%)^†^	73 (68.2%)*,^†^,^§^	<0.001
Peripheral oedema (%)	284 (23.6%)	36 (16.3%)	86 (19.5%)	120 (27.9%)*,^†^	42 (37.2%)*,^†^	<0.001
Diuretic use (%)	714 (58.9%)	87 (38.7%)	220 (49.9%)*	322 (74.2%)*,^†^	85 (75.9%)*,^†^	<0.001
Pacemaker/ICD (%)	401 (33.0%)	58 (25.7%)	131 (29.2%)	170 (39.4%)*,^†^	42 (38.5%)	<0.001
ACEi/ARB use (%)	702 (61.4%)	109 (50.9%)	251 (60.9%)	287 (69.2%)*	55 (53.9%)^§^	<0.001
Beta‐blocker use (%)	685 (59.8%)	120 (55.8%)	244 (59.1%)	255 (61.4%)	66 (64.1%)	0.430
Aldosterone receptor antagonist use (%)	243 (21.3%)	28 (13.1%)	68 (16.6%)	123 (29.6%)* ^,^ ^†^	24 (23.5%)	<0.001
Heart failure classification						
LVEF ≥ 50%	461 (37.7%)	106 (46.3%)	101 (42.8%)	123 (28.3%)*	41 (36.3%)	<0.001
LVEF = 41–49%	259 (21.2%)	61 (26.6%)	92 (20.6%)	88 (20.2%)	18 (15.9%)	
LVEF ≤ 40%	503 (41.1%)	62 (27.1%)	163 (36.5%)	224 (51.5%)*,^†^	54 (47.8%)*	

Values are presented as mean ± SD, median (IQR) or *n* (%).

ACEi, angiotensin‐converting enzyme inhibitor; ARB, angiotensin receptor blocker; eGFR, estimated glomerular filtration rate; ICD, implantable cardiac defibrillator; LVEF, left ventricular ejection fraction; NYHA, New York Heart Association.

* *P* < 0.05 vs. Group I.

^†^ *P* < 0.05 vs. Group II.

^§^ *P* < 0.05 vs. Group III.

The echocardiographic characteristics of the overall population are presented in Table 2. Patients with moderately or severely impaired renal function had larger LV, RV and left atrial dimensions, lower LV ejection fraction, more impaired RV systolic function and higher pulmonary arterial pressures than those with normal or mildly impaired renal function. In addition, patients with moderate or severe renal impairment had a larger tricuspid vena contracta width and tricuspid regurgitant volume and more frequently had significant mitral regurgitation when compared to individuals with normal or mildly impaired renal function.

**Table 2:** Echocardiographic characteristics

Variable	Total population (*n* = 1234)	Group 1: GFR ≥ 90 mL min^−1^ 1.73 m^−2^ (*n* = 230)	Group 2: GFR 60‐89 mL min^−1^ 1.73 m^−2^ (*n* = 451)	Group 3: GFR 30‐59 mL min^−1^ 1.73 m^−2^ (*n* = 439)	Group 4: GFR <30 mL min^−1^ 1.73 m^−2^ (*n* = 114)	*P* value
Left ventricle and atrium						
LV EDD (mm)	48.9 (±11.7)	46.3 (±10.5)	47.2 (±11.2)	51.3 (±12.3)* ^,^ ^†^	51.2 (±10.7)* ^,^ ^†^	<0.001
LV ESD (mm)	39.0 (±13.4)	35.8 (±11.7)	37.8 (±12.3)	41.4 (±14.9)* ^,^ ^†^	40.9 (±12.9)*	<0.001
LV EDV (ml/m^2^)	114 (82–169)	104 (75–151)	104 (77–144)	128 (87–209)* ^,^ ^†^	136 (103–192)* ^,^ ^†^	<0.001
LVEF (%)	43.9 (±15.8)	48.7 (±15.5)	45.4 (±15.6)	40.2 (±15.0)* ^,^ ^†^	42.5 (±16.5)*	<0.001
LA volume (mL)	92 (61–126)	74 (51–109)	89 (59–123)*	101 (66–131)*	98 (69–132)*	<0.001
Significant AS (%)	251 (21.2%)	35 (16.4%)	92 (21.2%)	98 (23.0%)	26 (24.1%)	0.225
Significant MR (%)	336 (27.4%)	42 (18.3%)	108 (24.2%)	144 (32.9%)* ^,^ ^†^	42 (37.2%)* ^,^ ^†^	<0.001
Right heart						
RV basal diameter, mm	45.6 (±8.6)	44.4 (±8.8)	44.9 (±8.1)	46.8 (±9.0)* ^,^ ^†^	46.2 (±7.8)	0.001
RV mid‐diameter, mm	35.3 (±9.0)	33.9 (±8.9)	34.6 (±8.9)	36.4 (±9.2)* ^,^ ^†^	36.5 (±8.4)	0.001
RV EDA, cm^2^	23.6 (18.5–29.8)	21.6 (17.7–28.4)	22.7 (18.1–28.2)	24.8 (19.2–31.5)* ^,^ ^†^	26.9 (20.5–31.3)* ^,^ ^†^	<0.001
RA area, cm^2^	25.7 (20.2–33.2)	23.5 (18.4–30.8)	25.8 (20.2–33.2)*	26.6 (20.8–34.9)*	26.5 (21.3–32.3)	0.010
TAPSE, mm	15.3 (±5.1)	16.1 (±5.2)	15.9 (±5.1)	14.6 (±4.9)* ^,^ ^†^	13.9 (±4.6)* ^,^ ^†^	<0.001
PASP, mmHg	43.0 (±16.9)	40.4 (±18.1)	41.9 (±16.4)	44.7 (±16.2)*	46.6 (±17.8)*	0.001
PASP > 40 mmHg (%)	618 (53.9%)	89 (42.6%)	212 (50.1%)	248 (60.3%)* ^,^ ^†^	69 (66.3%)* ^,^ ^†^	<0.001
eRAP, mm Hg	9.0 (±4.9)	9.2 (±4.7)	8.4 (±5.0)	9.2 (±4.9)^†^	10.1 (±4.8)	0.006
Tricuspid valve						
Moderate TR (%)	948 (76.9%)	178 (77.7%)	363 (80.5%)	328 (74.7%)	79 (69.9%)	0.054
Severe TR (%)	284 (23.1%)	51 (22.3%)	88 (19.5%)	111 (25.3%)	34 (30.1%)	0.054
TA diameter, mm	41.7 (±8.0)	40.1 (±7.9)	41.6 (±8.0)	42.5 (±8.1)*	42.5 (±7.8)	0.003
Vena contracta, mm	10.4 (±4.03)	9.7 (±4.2)	10.5 (±4.0)	10.6 (±3.9)*	11.2 (±4.2)*	0.006
EROA, mm^2^	64 (40–101)	56 (34–100)	64 (42–96)	67 (42–107)	64 (42–100)	0.536
RVol, mL/beat	60 (36–100)	52 (28–90)	60 (36–96)	66 (37–105)*	64 (42–114)*	0.007

Values are presented as mean ± SD, median (IQR) or *n* (%).

AS, aortic stenosis; EDA, end‐diastolic area; EDD, end‐diastolic diameter; EDV, end‐diastolic volume; EF, ejection fraction; eRAP, estimated right atrial pressure; EROA, effective regurgitant orifice area; ESD, end‐systolic diameter; LA, left atrial; LV, left ventricle; LVEF, left ventricular ejection fraction; MR, mitral regurgitation; PASP, pulmonary artery systolic pressure; RA, right atrial; RV, right ventricle; RVol, regurgitant volume; TA, tricuspid annulus; TAPSE, tricuspid annular plane systolic excursion; TR, tricuspid regurgitation.

* *P* < 0.05 vs. Group I.

^†^ *P* < 0.05 vs. Group II.

^§^ *P* < 0.05 vs. Group III.

### Association of echocardiographic parameters of TR severity with significant renal impairment

**Fig. 1:**
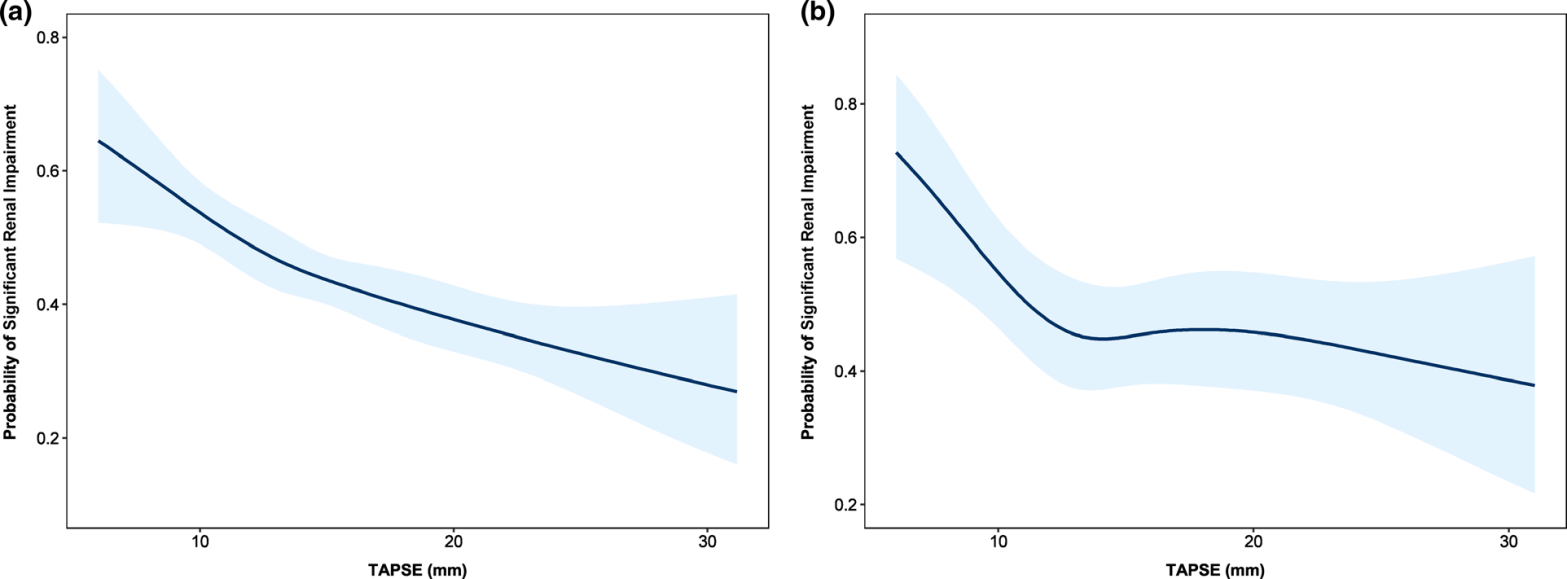
Spline curves demonstrating the probability of significant renal impairment (eGFR <60 mL min^−1^ 1.73 m^−2^) according to TAPSE in unadjusted (a) and adjusted models (b). The curve in panel a demonstrates the probability of significant renal impairment according to TAPSE measured at the time of index echocardiogram, with overlaid 95% confidence intervals displayed (shaded blue areas). The curve in panel b demonstrates the probability of significant renal impairment according to values of TAPSE, adjusted for age, diabetes mellitus, hypertension, ACEi/ARB use, diuretic use, aldosterone antagonist use, LV end‐diastolic volume, LV ejection fraction, the presence of significant MR, RV end‐diastolic area, tricuspid annulus diameter, TR regurgitant volume, estimated RAP and PASP. ACEi, angiotensin‐converting enzyme inhibitor; ARB, angiotensin receptor blocker; eGFR, estimated glomerular filtration rate; LV, left ventricle; MR, mitral regurgitation; PASP, pulmonary artery systolic pressure; RAP, right atrial pressure; RV, right ventricle; TAPSE, tricuspid annular plane systolic excursion; TR, tricuspid regurgitation.

To investigate the association between the pathophysiological mechanisms identified by echocardiography and significant renal impairment, univariable logistic regression analysis was performed, including clinical and echocardiographic variables known or postulated to be associated with significant renal impairment in patients with secondary TR [12, 13, 14]. On univariable analysis, age, diabetes mellitus, hypertension, angiotensin‐converting enzyme inhibitor/ angiotensin receptor blocker use, diuretic use, aldosterone antagonist use, LV ejection fraction and LV end‐diastolic volume were associated with significant renal impairment (Table S1). Of the parameters associated with TR severity, decreasing TAPSE, increasing TR vena contracta width, TR regurgitant volume, tricuspid annulus diameter, RV end‐diastolic area, estimated RA pressure and pulmonary artery systolic pressure were associated with the presence of significant renal impairment on univariable analysis. On multivariable logistic regression, following adjustment for important covariates, age, diabetes mellitus, diuretic use and LV end‐diastolic volume remained associated with significant renal impairment at the time of baseline echocardiography (Table 3). Of all the echocardiographic parameters related to TR severity, only TAPSE was associated with significant renal impairment in the multivariable model. Subsequently, spline curve analysis was performed to investigate the nature of the association between TAPSE and the probability of significant renal impairment at the time of echocardiography (Fig. 1). In the adjusted model (Fig. 1, Panel b), following a long plateau phase and no evidence of an association, there was a significant increase in the probability of significant renal impairment with values of TAPSE <14 mm. Values of TAPSE <14 mm were associated with the presence of significant renal impairment in the adjusted model (OR 1.49, 95% CI 1.11 to 1.99, *P* = 0.008).

**Table 3:** Multivariable logistic regression for parameters associated with significant renal impairment (eGFR < 60 mL min^−1^ 1.73 m^−2^) and severely impaired renal function (eGFR < 30 mL min^−1^ 1.73 m^−2^)

	Multivariable analysis for significant renal impairment (<60 mL min^−1^ 1.73 m^−2^)		Multivariable analysis for severe renal impairment (<30 mL min^−1^ 1.73 m^−2^)	
	OR (95% CI)	*P* value	OR (95% CI)	*P* value
Patient demographics and comorbidities				
Age, years	1.034 (1.021–1.047)	<0.001	1.001 (0.981–1.022)	0.914
Diabetes mellitus	1.922 (1.342–2.752)	<0.001	3.860 (2.352–6.336)	<0.001
Hypertension	1.372 (0.913–2.063)	0.128	2.518 (1.114–5.691)	0.026
ACEi/ARB use	0.917 (0.664–1.265)	0.597	0.412 (0.245–0.691)	<0.001
Diuretic use	2.339 (1.696–3.226)	<0.001	2.157 (1.164–3.997)	0.015
Aldosterone antagonist	1.266 (0.875–1.831)	0.211	0.656 (0.361–1.191)	0.166
Echocardiographic variables				
LV EDV, mL	1.004 (1.002–1.006)	0.001	1.004 (1.000–1.007)	0.028
LVEF, %	0.994 (0.984–1.005)	0.288	1.016 (0.998–1.034)	0.075
Significant MR	1.137 (0.827–1.564)	0.428	1.390 (0.834–2.317)	0.206
RV EDA, mm^2^	1.009 (0.995–1.024)	0.189	0.997 (0.969–1.025)	0.811
TA diameter, mm	0.997 (0.976–1.017)	0.739	0.985 (0.950–1.022)	0.433
TR RVol, mL	1.000 (0.997–1.003)	0.998	1.002 (0.998–1.007)	0.271
TAPSE, mm	0.963 (0.935–0.992)	0.012	0.944 (0.893–0.997)	0.038
Estimated RAP, mmHg	0.978 (0.947–1.010)	0.174	1.014 (0.961–1.070)	0.605
PASP, mmHg	1.006 (0.997–1.015)	0.217	1.001 (0.987–1.016)	0.854

ACEi, angiotensin‐converting enzyme inhibitor; ARB, angiotensin receptor blocker; EDA, end‐diastolic area; EDV, end‐diastolic volume; EF, ejection fraction; eRAP, estimated right atrial pressure; LV, left ventricle; LVEF, left ventricular ejection fraction; MR, mitral regurgitation; PASP, pulmonary artery systolic pressure; RV, right ventricle; RVol, regurgitant volume; TA, tricuspid annulus; TAPSE, tricuspid annular plane systolic excursion; TR, tricuspid regurgitation.

In a sensitivity multivariable logistic regression analysis, of the echocardiographic parameters associated with TR, only TAPSE was related to the probability of presenting with severe renal impairment (eGFR < 30 mL min ^−1^1.73 m^−2^) (Table 3). A further sensitivity analysis utilizing uni‐ and multivariable linear regression was performed to investigate the association between parameters related to TR severity and eGFR as a continuous variable (Table S2). Results consistent with those of the previous analyses were observed, with TAPSE being the only pathophysiological mechanism identifiable by echocardiography that was associated with eGFR after adjusting for potential confounders.

### Survival analysis

**Fig. 2:**
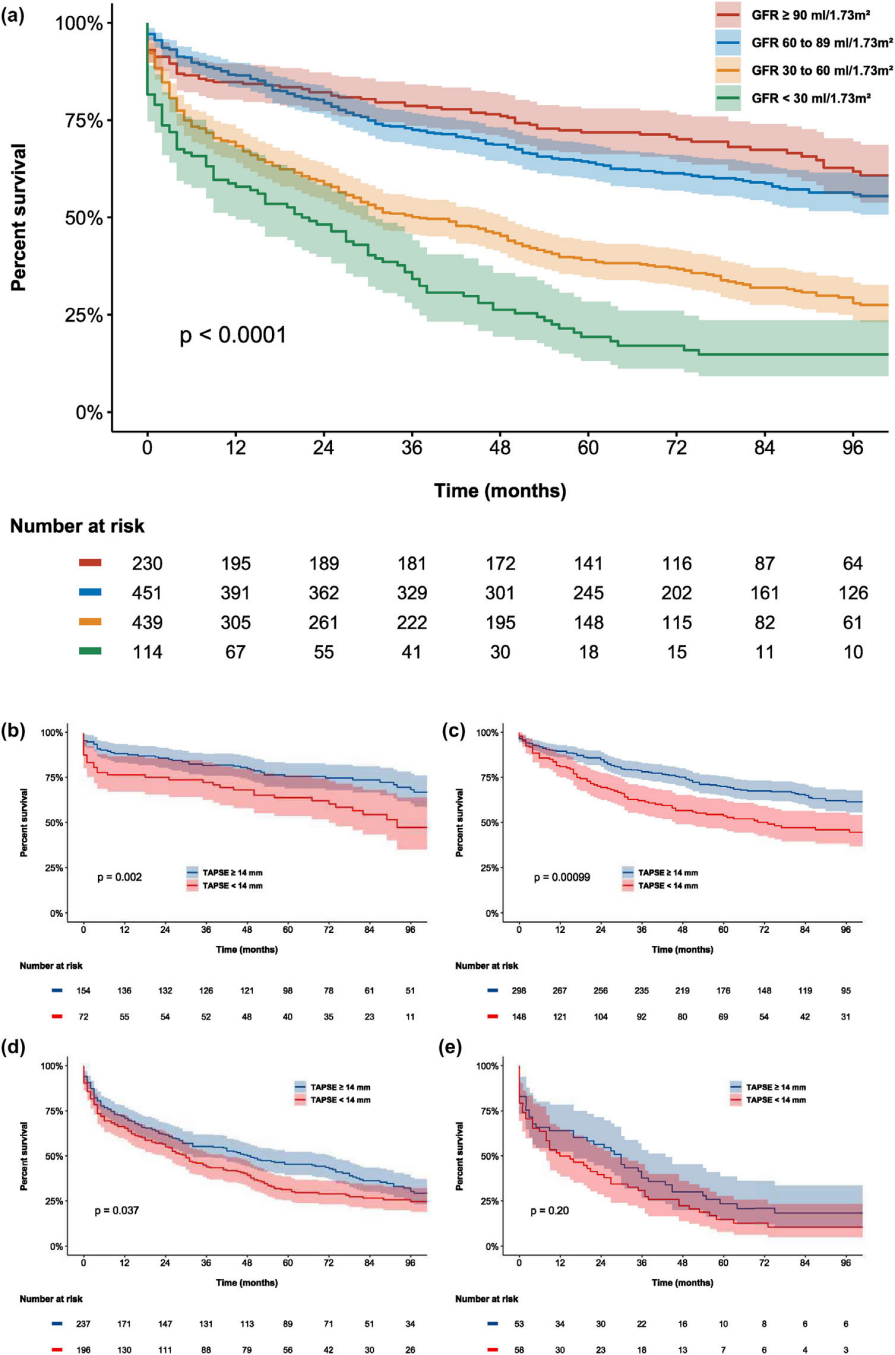
Kaplan–Meier estimates for all‐cause mortality stratified by renal function group and according to the presence of severe RV dysfunction (TAPSE < 14 mm). The Kaplan–Meier curves demonstrate reduced survival with worsening renal function (panel A) and the improved survival rates of patients with TAPSE ≥ 14 mm (blue line) compared to those with TAPSE <14 mm (red line) in renal function stage 1 (panel b), 2 (panel c) and 3 (panel d) CKD. For patients with severe renal impairment (stage 4 and 5 CKD, eGFR <30 mL min^−1^ 1.73 m^−2^), the presence of severe RV dysfunction did not portend a worse prognosis (panel e). CKD, chronic kidney disease; GFR, glomerular filtration rate; TAPSE, tricuspid annular plane systolic excursion.

Over a median follow‐up of 53 (interquartile range, 16–89) months, 692 patients (56%) died. The 1‐ and 5‐year cumulative survival rates were 77% and 53%, respectively, for the total population. The Kaplan–Meier analysis for all‐cause mortality demonstrated a significant reduction in survival for patients with worse renal function at 1 and 5 years (85% vs. 87% vs. 68% vs. 58% at 1 year, and 72% vs. 64% vs. 39% vs. 19% at 5 years, for stage 1, 2, 3 and 4‐5 CKD groups, respectively, *P* < 0.001) (Fig. 2A). In addition, the Kaplan–Meier survival analysis demonstrated that the presence of severe RV dysfunction was associated with a reduction in overall survival in the stage 1‐3 CKD groups, but not in stage 4‐5 CKD groups (Fig. 2B‐E).

## Discussion

In this study of 1234 patients with significant secondary TR, the prevalence of significant renal impairment (eGFR < 60 mL min^−1^ 1.73 m^−2^) was 45%. On multivariable analysis, age, diabetes mellitus, diuretic use and LV end‐diastolic volume were associated with significant renal impairment. Of all the pathophysiological mechanisms identified by echocardiography that are related to TR, only severe RV dysfunction (TAPSE < 14 mm) was independently associated with the presence of significant renal impairment. In addition, worsening renal function was associated with a significant reduction in survival at long‐term follow‐up. Severe RV dysfunction was associated with reduced overall survival in stage 1‐3 CKD groups, but not in stage 4‐5 CKD groups.

### Prevalence of renal dysfunction in moderate‐to‐severe TR

The prevalence of significant renal impairment (eGFR < 60 mL min^−1^ 1.73 m^−2^) in patients with significant secondary TR and heart failure with reduced ejection fraction (HFrEF) has previously been reported as 45–50%, in agreement with the results of the present study, which evaluated patients with significant secondary TR due to a variety of aetiologies [2]. This is in contrast to a recent study of 2380 patients with significant secondary TR of various aetiologies, where the reported prevalence of significant renal impairment was only 14%, although a specific definition of renal impairment was not provided [15].

### Association between echocardiographic parameters of TR severity and CKD

Although previous studies [2, 5] have clearly demonstrated an independent association between worse renal function and increasing grade of TR in patients with HFrEF, there has been minimal investigation into the possible mechanisms linking significant secondary TR and significant renal impairment. From a theoretical perspective, numerous echocardiographic parameters associated with the presence of significant TR could be directly related to increased central venous pressure and venous congestion and, consequently, renal impairment (i.e. increased TR volume, estimated RA pressure or RV dysfunction). In the present study of over 1,200 patients with significant secondary TR of various aetiologies, of the echocardiographic parameters associated with TR, we observed that only TAPSE was independently associated with significant renal impairment (eGFR < 60 mL min^−1^ 1.73 m^−2^) and severe renal impairment (eGFR < 30 mL min^−1^ 1.73 m^−2^). Moreover, in an adjusted non‐linear model, this relationship was only evident at values of TAPSE <14 mm (i.e. severe RV dysfunction), further strengthening the notion of a biologically plausible association. It is possible that previous associations observed between significant renal impairment and the grade of TR were actually indicative of the increased incidence of RV dysfunction observed with increasing TR severity. In addition, these findings are consistent with a previous study of 373 patients with HFrEF, where TAPSE ≤14 mm was independently associated with the presence of significant renal impairment [16]. However, the authors did not have access to important echocardiographic data pertaining to the severity of TR, so were unable to adjust for vital confounding variables in their analysis.

### Pathophysiological interactions between the right ventricle and kidney in significant secondary TR

**Fig. 3:**
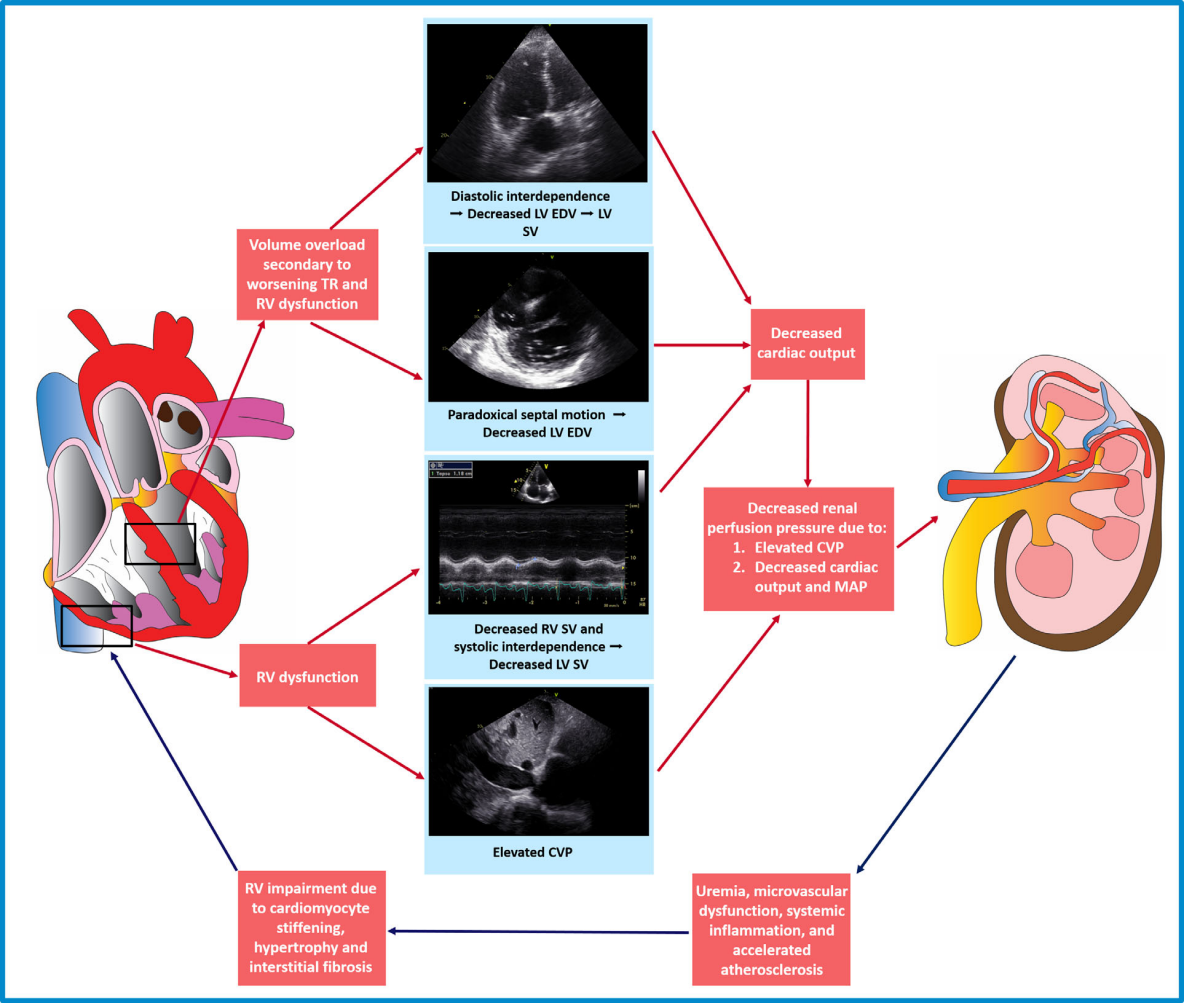
Pathophysiological interactions between the right ventricle and kidney in significant tricuspid regurgitation. CVP, central venous pressure; EDV, end‐diastolic volume; LV, left ventricle; MAP, mean arterial pressure; RV, right ventricle; SV, stroke volume; TR, tricuspid regurgitation.

Numerous pathophysiological interactions between the kidney and the volume‐overloaded right ventricle may explain the independent association observed between RV dysfunction and renal impairment in the present study (Fig. 3). Essentially, any haemodynamic change contributing to a reduction in transrenal perfusion pressure (determined by the difference between mean arterial pressure and central venous pressure) may lead to a reduction in eGFR [17]. In individuals with RV dysfunction, LV cardiac output may be reduced as a direct result of decreased RV cardiac output (as a series interaction) [18] and/or as a result of a reduction in ventricular systolic interdependence [19]. In addition, RV dysfunction may lead to RV remodelling and increased volume as a compensatory response to maintain adequate RV stroke volume (heterometric adaptation) [20]. Increased RV volume may then impair LV filling secondary to increased ventricular diastolic interdependence and/or paradoxical diastolic septal motion [19], further decreasing LV cardiac output and therefore mean arterial pressure.

In addition to these important interactions with LV function, adequate RV function is also necessary for maintaining a low central venous pressure [21]. In the presence of severe RV dysfunction, the central venous pressure may rise, resulting in a further reduction in renal perfusion pressure [21]. Indeed, in an experimental study of 17 normal human subjects, artificially increasing intra‐abdominal venous pressure to 20 mmHg resulted in a reduction in GFR of approximately 30% and of renal plasma flow by almost 25% [22]. Numerous additional animal and human studies have since confirmed the close relationship between elevated central venous pressure and significant renal impairment [17, 23], with several studies demonstrating that central venous pressure may be more important than forward cardiac output in modulating renal function [24, 25]. Interestingly, in the present study, no independent association between significant renal impairment and estimated RA pressure was observed, suggesting that the association with RV dysfunction may not be mediated by increased central venous pressure. However, the estimation of right atrial pressure on echocardiography through the evaluation of the inferior vena cava diameter and collapsibility may correlate less closely with invasively derived right atrial pressure in patients with significant TR [26]. Furthermore, estimated RA pressure may change acutely with alterations in volume or clinical status, whereas RV dysfunction may more accurately identify patients who are exposed to the cumulative effects of chronically elevated central venous pressure. In addition, estimated RA pressure may rise with even minimal exertion, which may not be captured on resting echocardiogram.

Importantly, pathophysiological consequences of renal impairment, including uraemia, microvascular dysfunction, inflammation, cytokine release and accelerated atherosclerosis, could directly result in progressive cardiomyocyte stiffening, hypertrophy and interstitial fibrosis, manifesting as worsening RV function [27, 28]. Indeed, these cardiorenal interactions may also potentially explain some of the association observed between RV dysfunction and renal impairment in the present study.

### Prognostic and clinical implications of renal impairment in secondary TR

Significant TR induces RV remodelling, characterized by progressive RV dilation and dysfunction [29]. In our study, we demonstrated that lower values of TAPSE are associated with worse renal function, which, in turn, may exacerbate the volume overload on the right ventricle and induce a vicious circle of progressive RV remodelling through a variety of mechanisms [30].

Interventions and therapies aiming to reduce the impact of potential causes of secondary TR may halt RV remodelling and also improve renal function. Significant left‐sided valvular heart disease and the consequent increase in pulmonary pressures are among the major determinants of secondary TR [31], and targeted interventions have shown a beneficial effect on renal function [32]. However, these beneficial effects on renal function have yet to be specifically linked with changes in TR or RV function.

Tricuspid valve interventions have the potential to reduce central venous pressure [33], halt the remodelling of the right ventricle, increase stroke volume [34], improve peripheral perfusion and, theoretically, permit the recovery of renal function. However, although Karam et al. demonstrated a positive impact of transcatheter tricuspid valve interventions on liver function in a cohort of 126 patients, no improvement in renal function was recorded during 6 months of follow‐up [35]. Nevertheless, they did not stratify their results according to pre‐procedural renal function and RV remodelling, factors that could logically impact on the likelihood of renal function recovery. Severe renal impairment (eGFR < 30 mL min^−1^ 1.73 m^−2^) may represent a degree of organ dysfunction that is too advanced to derive significant survival benefit from tricuspid valve interventions and the consequent RV reverse remodelling that may arise from the unloading of the right ventricle. Our results and the study by Karam et al. [35] may underline the importance of adequate risk stratification, screening and patient selection for tricuspid valve interventions, an assessment that should probably also include an evaluation of renal function.

### Limitations

This study is subject to the inherent limitations of a single‐centre, observational, retrospective design. While an independent association between RV dysfunction and significant renal impairment was observed, causality could not be established due to study design. The effects of tricuspid valve interventions on RV remodelling, renal function and the potential relationship with patient outcomes require further investigation.

## Conclusion

Of the pathophysiological mechanisms identified by echocardiography that are associated with significant secondary TR, only severe RV dysfunction (TAPSE < 14 mm) was independently associated with the presence of significant renal impairment. In addition, worse renal function according to CKD group was associated with a significant reduction in survival at long‐term follow‐up.

## Conflict of interest

The Department of Cardiology of the Leiden University Medical Center received research grants from Abbott Vascular, Bayer, BioVentrix, Medtronic, Biotronik, Boston Scientific, GE Healthcare and Edwards Lifesciences. Jeroen Bax and Nina Ajmone Marsan received speaking fees from Abbott Vascular. Victoria Delgado received speaker fees from Abbott Vascular, Medtronic, Edwards Lifesciences, MSD and GE Healthcare. The remaining authors have nothing to disclose.

## Funding

Steele C. Butcher received funding from the European Society of Cardiology (ESC Research Grant App000080404). This work was funded by an unrestricted research grant from Edwards Lifesciences (IISUSTHV2018017).

## Supporting information

**Table S1**. Univariable logistic regression for parameters associated with significant renal impairment (eGFR < 60 mL min^−1^ 1.73 m^−2^).
**Table S2**. Univariable and multivariable linear regression for estimated GFR.
**Figure S1**. Study flow chart.
**Figure S2**. Distribution of patients with significant secondary tricuspid regurgitation across groups of renal function for the overall population.
