# Supplementary material for: Renal function in patients with significant tricuspid regurgitation: pathophysiological mechanisms and prognostic implications
Source: J Intern Med. 2021 Jun 10;290(3):715–27. doi: 10.1111/joim.13312 (PMC8453518; doi:10.1111/joim.13312)
Supplement: Supplementary file 1 — Table S1. Univariable logistic regression for parameters associated with significant renal impairment (eGFR < 60 mL min−1 1.73 m−2). Table S2. Univariable and multivariable linear regression for estimated GFR. Figure S1. Study flow chart. Figure S2. Distribution of patients with significant secondary tricuspid regurgitation across groups of renal function for the overall population. [file JOIM-290-715-s001.docx]

**Supplementary Material:**

**Table S1: Univariable logistic regression for parameters associated with significant renal impairment (eGFR <60** **ml/min/1.73 m^2^)**

|  | **Univariable analysis** | |
| --- | --- | --- |
|  | **OR (95% CI)** | ***P* value** |
| **Patient demographics and comorbidities** | | |
| Age, years | 1.027 (1.017 to 1.036) | <0.001 |
| BMI, kg/m^2^ | 1.016 (0.986 to 1.047) | 0.299 |
| Male sex | 0.958 (0.766 to 1.199) | 0.709 |
| Diabetes mellitus | 2.559 (1.906 to 3.437) | <0.001 |
| Hypertension | 1.871 (1.381 to 2.534) | <0.001 |
| ACEi/ARB use | 1.444 (1.134 to 1.838) | 0.003 |
| Diuretic use | 3.424 (2.679 to 4.377) | <0.001 |
| Beta blocker use | 1.182 (0.932 to 1.499) | 0.169 |
| Aldosterone antagonist use | 2.185 (1.636 to 2.919) | <0.001 |
| Current smoking | 0.839 (0.653 to 1.078) | 0.170 |
| **Heart Failure Category** |  |  |
| LVEF ≥ 50% | **Reference** |  |
| LVEF = 41-49% | 1.255 (0.918 to 1.715) | 0.155 |
| LVEF ≤ 40% | 2.238 (1.726 to 2.900) | <0.001 |
| **Echocardiographic variables** | | |
| LV EDV, ml | 1.005 (1.003 to 1.007) | <0.001 |
| LV EF, % | 0.976 (0.969 to 0.983) | <0.001 |
| Stroke volume, ml | 1.000 (0.999 to 1.001) | 0.764 |
| Significant MR | 1.784 (1.385 to 2.297) | <0.001 |
| Significant AS | 1.241 (0.938 to 1.640) | 0.130 |
| RV EDA, mm^2^ | 1.020 (1.008 to 1.032) | 0.001 |
| TA diameter, mm | 1.022 (1.008 to 1.036) | 0.003 |
| TR EROA, mm^2^ | 1.001 (0.999 to 1.002) | 0.340 |
| TR RVol, ml | 1.003 (1.001 to 1.005) | 0.004 |
| TAPSE, mm | 0.940 (0.919 to 0.963) | <0.001 |
| Estimated RAP, mmHg | 1.030 (1.006 to 1.055) | 0.013 |
| PASP, mmHg | 1.013 (1.006 to 1.020) | <0.001 |

ACEi = angiotensin converting enzyme inhibitor; ARB = angiotensin receptor blocker; AS = aortic stenosis; BMI = body mass index; EDA = end-diastolic area; EDV = end-diastolic volume; EF = ejection fraction; eRAP = estimated right atrial pressure; EROA = effective regurgitant orifice area; LV = left ventricular; LVEF = left ventricular ejection fraction; MR = mitral regurgitation; PASP = pulmonary artery systolic pressure; RA = right atrial; RV = right ventricular; RVol = regurgitant volume; TA = tricuspid annulus; TAPSE = tricuspid annular plane systolic excursion; TR = tricuspid regurgitation.

**Table S2: Univariable and multivariable linear regression for estimated GFR**

|  | **Univariable analysis** | | | **Multivariable analysis** | |
| --- | --- | --- | --- | --- | --- |
|  | **B (95% CI)** | ***P* value** | **B (95% CI)** | | ***P* value** |
| **Patient demographics and comorbidities** | | | | | |
| Age | -0.541 (-0.669 to -0.414) | <0.001 | -0.609 (-0.750 to -0.467) | | <0.001 |
| Obesity | -3.526 (-9.182 to 2.130) | 0.221 |  | |  |
| Male sex | 0.392 (-2.998 to 3.782) | 0.821 |  | |  |
| Diabetes mellitus | -13.269 (-17.559 to -8.979) | <0.001 | -8.580 (-13.121 to -4.039) | | <0.001 |
| Hypertension | -10.994 (-15.369 to -6.620) | <0.001 | -6.173 (-11.225 to -1.120) | | 0.017 |
| ACEi/ARB use | -7.337 (-10.947 to -3.726) | <0.001 | -0.268 (-4.337 to 3.802) | | 0.897 |
| Diuretic use | -17.084 (-20.433 to -13.734) | <0.001 | -9.462 (-13.579 to -5.346) | | <0.001 |
| Beta blocker use | -3.152 (-6.753 to 0.448) | 0.086 |  | |  |
| Aldosterone antagonist use | -9.753 (-14.045 to -5.461) | <0.001 | -1.748 (-6.499 to 3.004) | | 0.471 |
|  | | | | | |
| LV EDV, ml | -0.069 (-0.093 to -0.045) | <0.001 | -0.052 (-0.080 to -0.025) | | <0.001 |
| LVEF, % | 0.345 (0.238 to 0.452) | <0.001 | 0.044 (-0.087 to 0.175) | | 0.511 |
| Significant MR | -8.455 (-12.766 to -4.144) | <0.001 | -4.147 (-8.187 to -0.107) | | 0.044 |
| RV EDA, mm^2^ | -0.193 (-0.31 to -0.072) | 0.002 | -0.112 (-0.265 to 0.042) | | 0.154 |
| TA diameter, mm | -0.323 (-0.534 to -0.113) | 0.003 | 0.077 (-0.178 to 0.332) | | 0.554 |
| TR vena contracta, mm | -0.655 (-1.084 to -0.227) | 0.003 |  | |  |
| TR EROA, mm^2^ | -0.002 (-0.028 to 0.023) | 0.852 |  | |  |
| TR RVol, ml | -0.048 (-0.081 to -0.016) | 0.003 | 0.002 (-0.032 to 0.036) | | 0.910 |
| TAPSE, mm | 0.982 (0.651 to 1.314) | <0.001 | 0.566 (0.211 to 0.921) | | 0.002 |
| eRAP, mmHg | -0.255 (-0.612-0.103) | 0.162 |  | |  |
| PASP, mmHg | -0.221 (-0.325 to -0.117) | <0.001 | -0.072 (-0.181 to 0.037) | | 0.194 |

ACEi = angiotensin converting enzyme inhibitor; ARB = angiotensin receptor blocker; EDA = end-diastolic area; EDV = end-diastolic volume; eRAP = estimated right atrial pressure; EROA = effective regurgitant orifice area; LV = left ventricular; LVEF = left ventricular ejection fraction; MR = mitral regurgitation; PASP = pulmonary artery systolic pressure; RV = right ventricular; RVol = regurgitant volume; TA = tricuspid annulus; TAPSE = tricuspid annular plane systolic excursion; TR = tricuspid regurgitation.

**Figure S1: Study Flow Chart**

**
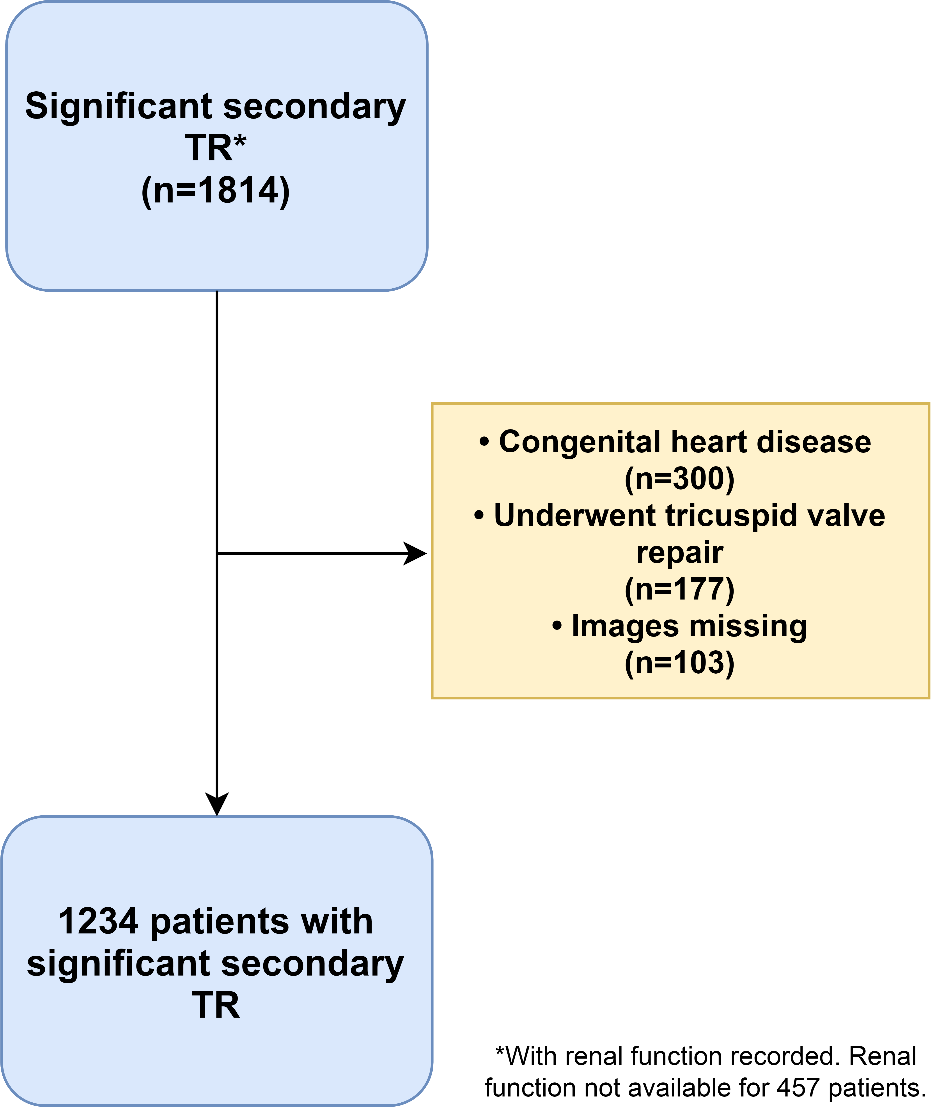
**

**Figure S2: Distribution of patients with significant secondary tricuspid regurgitation across groups of renal function for the overall population**

eGFR = estimated glomerular filtration rate
